# Phages Fight Back: Inactivation of the CRISPR-Cas Bacterial Immune System by Anti-CRISPR Proteins

**DOI:** 10.1371/journal.ppat.1005282

**Published:** 2016-01-07

**Authors:** Karen L. Maxwell

**Affiliations:** Donnelly Centre for Cellular and Biomolecular Research, University of Toronto, Toronto, Ontario, Canada; University of Kentucky, UNITED STATES

## Introduction

Phage infection poses a major threat to bacterial survival, and bacteria have evolved many mechanisms to protect against it. One such system is the CRISPR-Cas system, which utilizes sequence memory to protect bacteria from phage infection. CRISPR-Cas is a highly specific adaptive defense mechanism that protects against invasion by all mobile genetic elements, including phages, plasmids, and conjugative elements [1,2]. CRISPRs were first recognized in *Escherichia coli* in 1987 [3] and have since been identified in the genomes of approximately 48% of eubacteria and 95% of archaea [4]. Their widespread occurrence highlights the important role they play in the evolution of microbial and phage genomes.

CRISPR-Cas systems are composed of clustered regularly interspaced short palindromic repeats (CRISPR) separated by short “spacer” sequences, and CRISPR-associated (*cas*) genes. CRISPR systems are classified according to their gene complement and mechanism of action [2]. There are several different types (I, II, III) and subtypes (e.g., I-E, I-F) of CRISPR-Cas systems, most of which target DNA. The CRISPR system functions by incorporating one or more CRISPR spacers at the leader end of a CRISPR locus when a novel phage or other mobile genetic element infects the bacterial cell. These spacers are derived from the DNA of the invading phage, and their introduction into the CRISPR locus provides immunity to further infection by that phage. This adaptive immunity serves to protect the bacterial progeny; if the same phage is encountered in the future, the CRISPR-Cas system will be activated and the foreign genome destroyed. In turn, the phages mutate to evade CRISPR targeting, thereby creating a constant evolutionary battle between them and the bacteria they infect.

## *Pseudomonas* Phages Encode Proteins That Inactivate the CRISPR-Cas System

Our group recently discovered that some *Pseudomonas aeruginosa* phages encode proteins that inactivate the CRISPR-Cas system. A total of five distinct anti-CRISPR protein families that inhibit the type I-F system [5] and four protein families that inhibit the type I-E system [6] were identified. The genes encoding these proteins were found in a closely related group of Mu-like phages, positioned between conserved genes encoding the head protease/scaffold and homologues of the phage Mu gpG protein. All of the phages that carried this anti-CRISPR region also possessed a gene immediately downstream of the anti-CRISPR genes, encoding a helix-turn-helix protein that likely acts as a transcriptional regulator of the operon, and a very similar DNA sequence upstream that appears to be a promoter, with -10 and -35 transcriptional initiation sequences. Together, this forms an independent module that allows the expression of the anti-CRISPR genes from the prophage as well as during a lytic phage infection.

The anti-CRISPR operon was found in the same genomic position in a variety of related *Pseudomonas* phages. However, the complement of particular anti-CRISPR genes that comprised the locus varied between phages. For example, of 24 related phages that possess an anti-CRISPR operon, 15 encoded both type I-E and type I-F anti-CRISPR genes, one encoded only type I-E, and eight encoded only type I-F [6]. Collectively, these phages provided a total of nine distinct arrangements of various type I-E and I-F anti-CRISPR genes, illustrating that these genes have re-assorted multiple times through horizontal gene transfer in a “mix and match” manner. While it is difficult to trace the evolutionary origins of the anti-CRISPR proteins (as they have few sequence homologues), their frequent occurrence in *Pseudomonas* phages implies that they provide a significant evolutionary advantage.

## Anti-CRISPR Proteins Function by a Variety of Mechanisms

**Fig 1:**
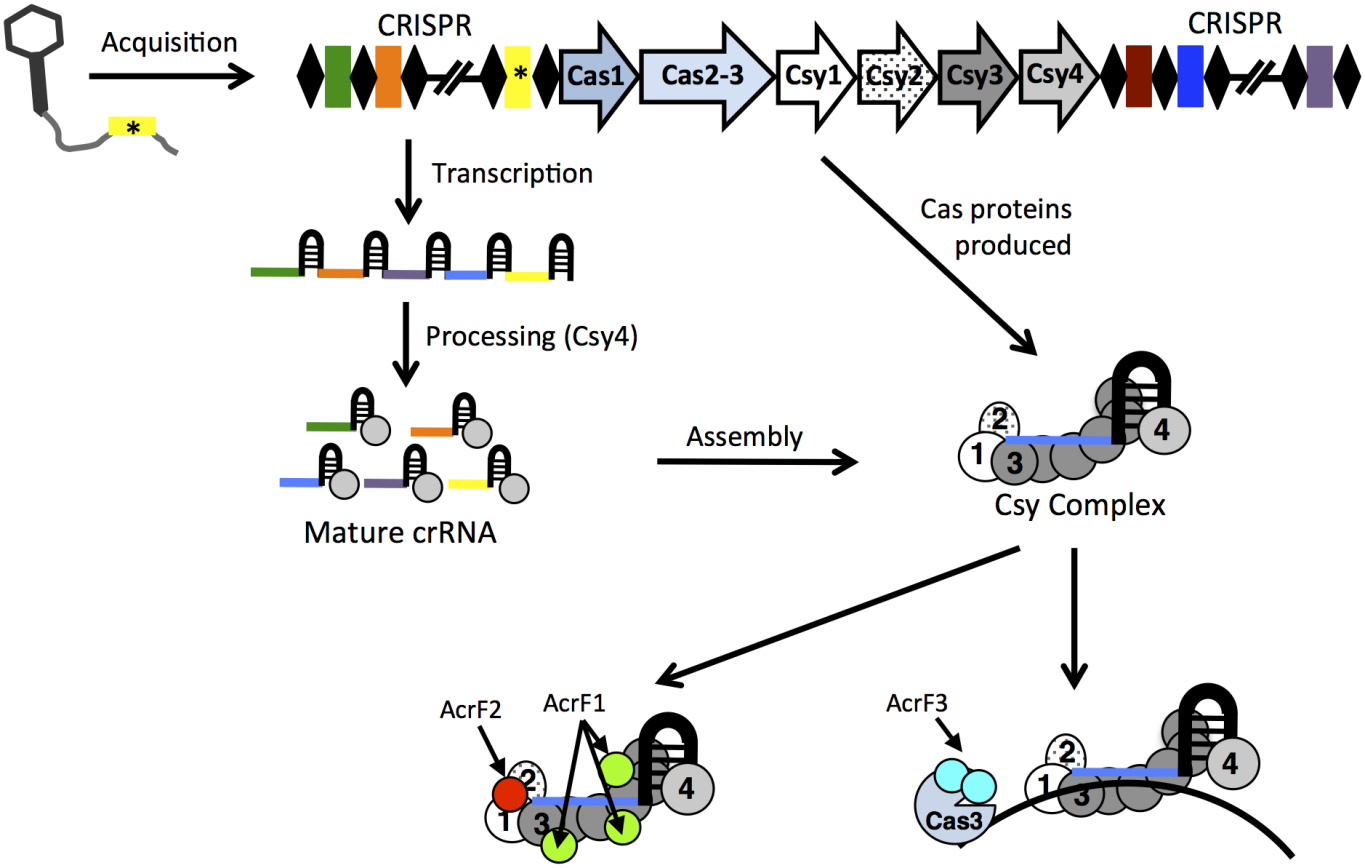
A model of the mechanisms of action of three anti-CRISPR proteins. The type I-F CRISPR loci consist of direct repeat sequences (black diamonds) separated by unique spacer sequences (colored rectangles) that were derived from foreign mobile genetic elements. During the acquisition stage, a piece of DNA from an invading phage or other mobile element is captured and inserted as a spacer into a CRISPR array (yellow rectangle). Next, the CRISPR-associated (Cas) proteins are produced, and the CRISPR loci are transcribed into long pre-CRISPR-RNAs. The Csy4 endonuclease then cleaves the repeat sequences to yield mature CRISPR-RNAs (crRNAs), each containing a single spacer sequence (colored lines). The crRNA-Csy4 complex then interacts with Csy1, Csy2, and Csy3 to form the surveillance complex. AcrF1 and AcrF2 directly interact with this complex and prevent DNA binding. By contrast, AcrF3 binds to the Cas3 helicase–nuclease protein and blocks its recruitment.

The activity of the type I-F CRISPR-Cas system against phages is divided into three stages (Fig 1). First, the Cas1 and Cas2 proteins recognize a target sequence within the invading phage genome and incorporate it into the CRISPR array as a spacer. Next, the CRISPR array is transcribed into a long pre-CRISPR-RNA (crRNA) and cleaved at the repeat sequences to yield mature crRNAs that provide complementary base pairing with invading foreign DNA. This cleavage is mediated by the Csy4 protein endonuclease, which remains bound to the 3’ end of the crRNA [7]. Finally, the crRNA-Csy4 complex interacts with the Csy1, Csy2, and Csy3 proteins to form the Csy complex [8]. This complex surveys the bacterial cell and binds to invading foreign DNA that is complementary to the crRNA. Once bound to DNA, the Csy complex recruits the Cas3 helicase–nuclease protein, which mediates target DNA degradation [9,10].

The mechanisms of action of three different type I-F anti-CRISPR proteins have been characterized and were shown to be unique [11]. In the Csy complex, a Csy1-Csy2 heterodimer is bound at the 5’ end of the crRNA, a Csy4 monomer is bound at the 3’ end, and six Csy3 subunits are bound along the RNA spacer [7,12]. As shown in Fig 1, AcrF1 and AcrF2 interact directly with this complex; both proteins block DNA-binding when bound to the Csy complex before it interacts with the target DNA. AcrF2 bound to the Csy1-Csy2 heterodimer with a stoichiometry of one anti-CRISPR molecule per complex. This inhibits the activity of the CRISPR-Cas system by sterically blocking the 5’ end of the crRNA and competing with DNA for the overlapping binding site. AcrF1 was shown to bind along the full length of the Csy3 “spine” of the complex, with a stoichiometry of three molecules per Csy complex. In contrast with AcrF2, AcrF1 could bind the complex in the presence of bound DNA, suggesting that this anti-CRISPR functions through an allosteric mechanism. Thus, while AcrF1 and AcrF2 both bind the Csy complex, their mechanisms of activity are distinct; they bind different Csy complex subunits with different stoichiometry and utilize steric versus allosteric DNA occlusion mechanisms. The third anti-CRISPR protein, AcrF3, was found to interact directly with the Cas3 helicase–nuclease protein and thereby blocks its recruitment to the Csy complex (Fig 1). Thus, consistent with the diverse protein sequences, the mechanisms of activity of these anti-CRISPR proteins imply independent evolutionary pathways, despite their common genomic position in closely related *P*. *aeruginosa* phage genomes.

## Anti-CRISPRs Convert the CRISPR-Cas System into a Transcriptional Repressor

A significant feature of the CRISPR-Cas system is that it can be targeted to any DNA sequence, allowing the system to be engineered for programmable transcriptional repression. It was shown that by combining a deletion of the *E*. *coli* type I-E *cas3* helicase–nuclease gene with the introduction of CRISPR arrays targeting specific gene promoter regions, both endogenous and heterologous genes could be silenced [13]. In this system, the Csy complex stably bound to the DNA target sequence. In the absence of Cas3 nuclease activity, the bound complex was able to physically block access of RNA polymerase. Anti-CRISPR AcrF3, which inhibits the activity of Cas3, provides a similar mechanism for the control of bacterial gene expression. When AcrF3 was expressed in vivo from a prophage along with a plasmid that encoded a crRNA targeting the promoter of a gene required for production of the blue-green pyocyanin molecule (*phzM*), it repressed transcription [11]. These cultures showed a complete lack of pigment production, similar to that observed for a strain lacking *cas3*. This demonstrates that prophages have the ability to exploit the organism’s native CRISPR-Cas system for transcriptional regulation. This ability to control the activity of the type I-F CRISPR-Cas system via the expression of anti-CRISPR proteins provides a novel strategy for prophages to regulate bacterial gene expression and control bacterial phenotypes.

## Anti-CRISPR Genes Are Found in a Variety of Mobile Elements

Bacterial CRISPR-Cas systems target and destroy foreign DNA from all mobile genetic elements, including phages, plasmids, transposons, and pathogenicity islands. Like phages, the fitness of each of these mobile genetic elements would be increased if they were able to inactivate the CRISPR-Cas systems of bacteria upon invasion. Consistent with this, a number of anti-CRISPR homologues are found in various *Pseudomonas* strains within genome regions that are not phage-associated. These regions include several that are likely mobile elements, as indicated by the presence of genes encoding proteins that are involved in DNA transfer and conjugation [5,6]. Three of these non-phage-encoded anti-CRISPRs were shown to be active—two type I-E and one type I-F—with one region encoding both a type I-E and a type I-F anti-CRISPR protein [6]. These anti-CRISPR genes likely increase the fitness for the inter-strain transfer of these mobile elements by inactivating the CRISPR-Cas system of the recipient strain.

Anti-CRISPR proteins in mobile genetic elements may play important roles in increasing the virulence of bacterial strains. For example, an anti-CRISPR homologue is found in an active pathogenicity island of a highly virulent *P*. *aeruginosa* clinical isolate that is probably transferred between *P*. *aeruginosa* by conjugation [14]. This pathogenicity island contains 100% identity matches to CRISPR spacers in a variety of *P*. *aeruginosa* strains, indicating that it would be targeted and destroyed by the CRISPR system in these strains [15] without the protection provided by the anti-CRISPR protein. The discovery of anti-CRISPR genes in a variety of mobile genetic elements suggests that they may play significant roles in lateral gene transfer events by allowing incoming foreign DNA to bypass the CRISPR-Cas system.

## Conclusion

In the evolutionary arms race between phages and bacteria, where phages are constantly evolving to overcome bacterial defense systems, phages have developed a means by which they inactivate the CRISPR-Cas system. While anti-CRISPRs have thus far only been identified in association with the type I-E and I-F CRISPR systems in *Pseudomonas*, their diverse protein sequences and mechanisms of action, coupled with the strong selection imposed by the virus–host arms race, suggest that there is likely an abundance of anti-CRISPR proteins yet to be discovered.
